# Impact of the *MTHFR C677T* polymorphism on blood pressure and related central haemodynamic parameters in healthy adults

**DOI:** 10.1111/jhn.13061

**Published:** 2022-07-27

**Authors:** Martina Rooney, Catherine F. Hughes, J. J. Strain, Michelle Clements, Helene McNulty, Mary Ward

**Affiliations:** ^1^ Nutrition Innovation Centre for Food and Health (NICHE), School of Biomedical Sciences Ulster University Coleraine Northern Ireland

**Keywords:** blood pressure, haemodynamics, hypertension, methylenetetrahydrofolate reductase, personalised nutrition, riboflavin

## Abstract

**Background:**

The C677T polymorphism in the gene‐encoding methylenetetrahydrofolate reductase (MTHFR) is associated with an increased risk of hypertension and cardiovascular disease. Riboflavin, the MTHFR cofactor, is an important modulator of blood pressure (BP) in adults homozygous for this polymorphism (TT genotype). The effect of this genetic variant on BP and related central haemodynamic parameters in healthy adults has not been previously investigated and was examined in this study.

**Methods:**

Brachial BP, central BP and pulse wave velocity (PWV, SphygmoCor XCEL) were measured in adults aged 18–65 years prescreened for *MTHFR* genotype. Riboflavin status was assessed using the erythrocyte glutathione reductase activation coefficient assay.

**Results:**

Two hundred and forty‐two adults with the *MTHFR* 677TT genotype and age‐matched non‐TT (CC/CT) genotype controls were identified from a total cohort of 2546 adults prescreened for *MTHFR* genotype. The TT genotype was found to be an independent determinant of hypertension (*p* = 0.010), along with low‐riboflavin status (*p* = 0.002). Brachial systolic and diastolic BP were higher in TT versus non‐TT adults by 5.5 ± 1.2 and 2.4 ± 0.9 mmHg, respectively (both *p* < 0.001). A stronger phenotype was observed in women, with an almost 10 mmHg difference in mean systolic BP in TT versus non‐TT genotype groups: 134.9 (95% confidence interval [CI] 132.1–137.6) versus 125.2 (95% CI 122.3–128.0) mmHg; *p* < 0.001. In addition, PWV was faster in women with the TT genotype (*p* = 0.043).

**Conclusion:**

This study provides the first evidence that brachial and central BP are significantly higher in adults with the variant *MTHFR* 677TT genotype and that the BP phenotype is more pronounced in women.

## Key points

This is the first study to show that central blood pressure is significantly higher in *younger* adults with the variant *MTHFR* 677TT genotype compared to those with the CC/CT genotypes providing further evidence *for the role* of this genetic risk factor in hypertension. Furthermore, the BP phenotype and pulse wave velocity appear to be more pronounced in women compared to men. Riboflavin, the cofactor for the MTHFR enzyme, was found to be an important modulator of blood pressure, with suboptimal riboflavin status almost doubling the risk of hypertension. These data have relevance for public health given that riboflavin has been shown to lower blood pressure specifically in individuals with the *MTHFR* TT genotype. Given the high prevalence of the *MTHFR* C677T polymorphism globally, these findings have important implications for the prevention and treatment of hypertension in adults worldwide with this genetic risk factor.

## Graphical abstract

When stratified by MTHFR genotype women with TT had a systolic blood pressure of almost 10 mmHg higher than their non‐TT counterparts. Furthermore, the TT genotype was associated with higher brachial Mean Arterial Pressure and Pulse Pressure in women.

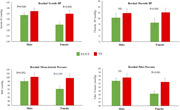

## INTRODUCTION

Hypertension continues to be a leading contributor to global mortality,^1^ with the prevalence estimated to have doubled between 1990 and 2019.^2^ Despite a trend towards improved blood pressure (BP) control in high‐income countries, control rates remain at 37% in the United Kingdom compared to approximately 50% in the United States and up to 69% in Canada, suggesting that other factors which are currently not being targeted, are at play.^3^ Genome‐wide association studies have identified a number of genetic loci associated with hypertension, including a locus close to the gene encoding the folate‐metabolising enzyme, methylenetetrahydrofolate reductase (MTHFR).^4^, ^5^ In addition, strong evidence from epidemiological and clinical studies shows that the common C677T polymorphism in this gene is associated with a 24–87% increased risk of hypertension and up to 40% increased risk for cardiovascular disease (CVD), particularly stroke.^6^ Of note, however, randomised controlled trials (RCTs) from this centre conducted in premature CVD patients and in hypertensive adults without overt CVD indicate that this phenotype can be significantly modulated by intervention with low‐dose riboflavin, the MTHFR cofactor.^7^, ^8^, ^9^ The influence of other factors such as age and sex on this phenotype is unknown but is relevant given that the homozygosity for the polymorphism (TT genotype) affects 2–32% of the global population^10^ and 10–12% of the UK and Irish populations.^11^ Recent observational analysis of 6076 adults demonstrated that carrying the variant TT genotype combined with riboflavin deficiency (prevalent in 30% of the cohort) tripled the likelihood of being classed as hypertensive (systolic BP ≥140 and/or diastolic BP ≥ 90 mmHg).^11^

To date, the evidence linking this polymorphism with BP is based mainly on brachial BP as the primary outcome. Central pressure and haemodynamic parameters are additional and potentially superior prognostic markers for CVD risk compared to clinic BP, as surrogate markers of large‐artery stiffness.^12^, ^13^ Central haemodynamic parameters can be noninvasively, reliably measured and have been widely reported in clinical studies; their use, however, in nutrition research is limited. The influence of the *MTHFR* genotype on central BP and haemodynamics has been confined to a small number of studies in specific patient cohorts, with limited data reported.^14^, ^15^, ^16^ Thus, a comprehensive profile of central haemodynamic parameters in healthy adults stratified by *MTHFR* genotype remains to be investigated.

Male sex is a well‐established risk factor for both hypertension and CVD. Hypertension‐related mortality is however reported to be higher in women compared to men across all age groups,^17^ a finding that may be explained, in part, by the steeper BP trajectories reported in women aged 30 years and persisting throughout the life course.^18^ Furthermore, cardiovascular physiology appears to be influenced by sex, and male–female differences in response to hypertension treatment have been reported.^19^ There is also some evidence suggesting sex differences in the influence of genetic variance on BP, with reports that genes are differentially expressed or contribute differently to diseases in men versus women.^20^ Differential regulation of genes may have important ramifications for health outcomes of pregnancy, with an estimated 10–15% of pregnancies affected by hypertension, which can lead to serious hypertensive disorders of pregnancy, including pre‐eclampsia.^6^ Although the *MTHFR* 677TT genotype has been linked with an increased risk of hypertension in pregnancy,^6^, ^21^ the effect of sex on the role of this common polymorphism in BP has been largely ignored to date.

The primary aim of this observational study, therefore, was to investigate BP and related central haemodynamic parameters in healthy adults aged 18–65 years stratified by the *MTHFR* C677T genotype, and the secondary aim was to consider the effect of sex on this association.

## METHODS

### Study design and participants

**Figure 1:**
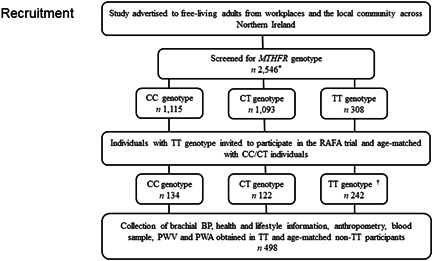
Flow chart of study population. ^*^30 swabs deemed unviable and no genotype result returned. ^†^Lost to follow‐up; uncontactable (*n =* 40); declined to participate (*n =* 26). CC (wild type), CT (heterozygous), TT (homozygous) genotypes for the 677 C → T polymorphism in *MTHFR*. BP, blood pressure; *MTHFR*, methylenetetrahydrofolate reductase; PWA, pulse wave analysis; PWV, pulse wave velocity; RAFA, Riboflavin And Folic Acid trial.

This study consisted of an observational investigation of brachial, or office, BP, central BP, pulse wave analysis (PWA) and pulse wave velocity (PWV) in healthy adults stratified by the *MTHFR* C677T genotype. Apparently healthy adults aged 18 years and older were recruited from workplaces and the wider community across Northern Ireland as part of the Riboflavin And Folic Acid (RAFA) study. The study has been registered at ClinicalTrials.gov (identifier NCT04948086), and ethical approval was granted by the Ulster University Research and Ethics Committee (UREC/11/0081). All participants provided written informed consent. Inclusion criteria were individuals aged 18 years and older and prescreened for the *MTHFR* genotype, and those who were identified as B‐vitamin supplement users, were pregnant or planning a pregnancy were excluded. DNA from 2546 participants was collected using buccal swabs and screened for the *MTHFR* C677T genotype at LGC Genomics using KASP technology. The study design is shown in Figure 1.

### BP measurements

Brachial BP was assessed using an Omron 705IT BP monitor and appropriately sized cuff (Cardiac Services, Belfast, UK). A fully trained researcher conducted the measurement consistent with a standard operating procedure and in accordance with NICE Guidelines for Hypertension in the United Kingdom.^22^ Briefly, after 10 min at rest, with the participant seated and the arm resting on a table, the reference arm was identified (the arm with the highest BP), and two BP readings taken 5 min apart were used to determine mean BP. If a difference of >5 mmHg was observed between readings, subsequent readings to a maximum of six were taken. Pulse pressure (PP) was calculated as systolic BP minus diastolic BP (mmHg) and mean arterial pressure (MAP) as 1/3 systolic BP plus 2/3 diastolic BP (mmHg).

### PWA and PWV

PWA and PWV were assessed noninvasively using the SphygmoCor XCEL device (AtCor Medical, NSW, Sydney, Australia) with the participant in the supine position on a flat examination couch after 5–10 min at rest. Speaking was avoided during the measurements, and participants were asked to abstain from caffeine, smoking, exercise and alcohol for 4 h before the assessment. The same researcher conducted all the measurements.

The central pressure waveform was derived from cuff pulsations recorded at the brachial artery. A brachial cuff was initially inflated to measure brachial systolic and diastolic BP and was reinflated to a subdiastolic pressure to acquire a volumetric displacement signal that automatically captured the PWA waveform for 5 s. A generalised transfer function, built in the manufacturer's software, calculated the aortic waveform. Augmentation pressure was calculated as the difference between the early and late systolic peaks of the estimated central pressure waveform. Augmentation index (AIx), normalised to a heart rate of 75 bpm by the internal software, was calculated as the augmentation pressure as a percentage of central PP. PP amplification was determined as the difference between central PP and brachial PP, and PP ratio was the brachial PP divided by the central PP.

Carotid‐femoral PWV was obtained by simultaneous acquisition of the carotid pulse by applanation tonometry (high‐fidelity transducer, Millar Instruments, Houston, Texas, USA) and the femoral pulse by volumetric displacement within a cuff placed around the upper thigh. PWV was calculated as the distance travelled over the time taken. Distance between the recording sites, in millimetres, was calculated using the subtraction method, that is, the distance between the suprasternal notch and the carotid pulse palpation point subtracted from the distance between the suprasternal notch and the proximal edge of the femoral cuff, using a nonstretch measuring tape. Transit time was determined using the ‘foot‐to‐foot’ method. Calibration was performed using the systolic and diastolic BP obtained by the brachial cuff in PWA.

### Questionnaire and anthropometric measurements

Details of the participant's health, including medications and family history of CVD, were obtained. Dietary information on habitual intake of specified foods (sources of B‐vitamins, including milk and fortified breakfast cereals) was collected using a researcher‐assisted food frequency questionnaire, previously validated for B‐vitamin intake against B‐vitamin biomarkers.^23^ Participants also had their weight (kg; SECA 770 scales), height (m) and waist circumference (cm) measured.

### Blood sampling and laboratory analysis

A 25‐ml nonfasting blood sample was collected from participants during the appointment by a trained phlebotomist into four separate blood collection tubes: 9‐ and 4‐ml EDTA vacutainers and 8‐ and 4‐ml serum vacutainers as described elsewhere.^24^ All blood tubes were placed on ice packs, processed within 4 h and stored at −80°C for batch analysis at the end of the study. Riboflavin status was determined at Ulster University by erythrocyte glutathione reductase activation coefficient (EGRac) assay. Oxidised glutathione was added and converted to reduced glutathione using NADPH as the reducing agent. This conversion was catalysed by glutathione reductase and mediated by flavin adenine dinucleotide (FAD), with the rate of absorbance measurable at a wavelength of 340 nm. EGRac was calculated by comparing the rate of absorbance change with added FAD compared to the rate of absorbance change without added FAD, with an EGRac ratio ≤1.26 indicating optimal riboflavin status, >1.26–<1.4 indicating suboptimal status and ≥1.4 signifying riboflavin deficiency.^25^ Analysis was conducted on a Daytona+ clinical chemistry analyser (Randox Laboratories Ltd, Antrim, Northern Ireland). Quality controls (QCs) were provided by repeated analysis of pooled samples covering a wide range of values. The inter‐assay variation was 2% for the high‐riboflavin QC and 3% for the low‐riboflavin QC.

### Power calculation

An estimation of sample size was based on observational differences in mean value and variability of 24‐h ambulatory BP between the *MTHFR* genotype groups from research previously conducted at this centre (McMahon et al., unpublished). An estimated sample size per group (i.e., 245 individuals with the TT genotype and 245 with the non‐TT group) was estimated with a power of 80% and *α =* 0.05 using G* power.^26^

### Statistical analysis

All statistical analysis was performed using SPSS Statistical Package for Social Sciences (version 25.0). Variables were tested for normality before analysis, and data were log transformed before analysis where appropriate. Differences between general characteristics were analysed using independent samples *t* tests. For outcome measures, differences between groups (non‐TT vs. TT) for continuous data were analysed by ANCOVA, controlling for age, sex, body mass index (BMI), use of antihypertensive medications and consumption of fortified breakfast cereals. Differences between categorical groups were analysed by *χ* ^2^ analysis. Logistic regression analysis was performed to examine the association between *MTHFR* genotype and the risk of hypertension after adjustment for established risk factors. *p* < 0.05 was considered statistically significant.

## RESULTS

### General characteristics

The study was advertised to an estimated 11,000 free‐living individuals from workplaces and the local community across Northern Ireland, from which 2546 individuals were recruited and provided a buccal swab to collect DNA to enable screening for the *MTHFR* genotype. This analysis yielded a prevalence of 44.3% with the CC genotype, 43.4% with the CT genotype and 12.2% with the TT genotype for the C677T polymorphism. Of those identified (*n =* 308) as having the TT genotype, 242 agreed to participate in this observational study (40 individuals with the TT genotype were uncontactable, and 26 declined to participate in the study) and were age matched with 134 CC and 122 CT genotype adults, yielding a total available sample of 498 study participants (Figure 1). The general characteristics of the *MTHFR* genotype are presented in Table 1. Participants had a mean age of 45.6 years and mean BMI in the overweight category, with 22.7% classified as normal weight, 43% as overweight and 34.3% as obese. The majority of participants (>90%) were regular milk consumers and reported to consume B‐vitamin‐fortified foods (>70%). There was a higher prevalence of hypertension (BP ≥140/90 mmHg) in those with the TT genotype. Also, despite similar rates of antihypertensive medication use across genotype groups, the TT genotype group was less likely to achieve BP control on treatment, albeit this failed to reach statistical significance. Riboflavin status, determined by the functional biomarker EGRac, was similar across the groups. Overall, the riboflavin status of 48.0% of the cohort was classed as optimal, 22.2% as suboptimal and 29.8% as deficient. There were no other significant differences in general characteristics between genotype groups. A detailed breakdown of antihypertensive drug use and drug combinations by genotype group is provided in Supporting Information, Table 1.

**Table 1:** General characteristics of study population by *MTHFR* genotype (*n =* 498)

	*MTHFR* genotype		*p*‐value*
	Non‐TT (*n* = 256)	TT (*n* = 242)	
Age (years)	45.5 (9.8)	44.8 (11.1)	0.311
Male sex *n* (%)	162 (63)	138 (57)	0.182
BMI (kg/m^2^)	28.9 (5.3)	29.1 (5.7)	0.902
Diabetes mellitus *n* (%)	3 (1)	9 (4)	0.119
Smoker *n* (%)	28 (11)	32 (13)	0.519
Alcohol consumer *n* (%)	192 (75)	195 (81)	0.165
Fortified food consumer^a^ *n* (%)	197 (77)	172 (71)	0.213
Milk consumer *n* (%)	233 (91)	227 (94)	0.317
Family history CVD *n* (%)	119 (47)	89 (37)	0.035
Statin use *n* (%)	20 (8)	15 (6)	0.597
Hypertension^b^ *n* (%)	76 (30)	97 (40)	0.019
Antihypertensive medication use *n* (%)	41 (16)	35 (15)	0.721
Treated and controlled^c^	26 (63)	16 (46)	0.188
*Riboflavin status*			
EGRac (biomarker)	1.33 (0.17)	1.36 (0.17)	0.057
Optimal (<1.26) *n* (%)	126 (52)	103 (44)	0.203
Suboptimal (1.26–1.40) *n* (%)	52 (21)	54 (23)	
Deficient (≥1.40) *n* (%)	65 (27)	77 (33)	

*Note*: Values are mean (SD) unless otherwise stated.

Abbreviations: BMI, body mass index; CVD, cardiovascular disease; EGRac, erythrocyte glutathione reductase activation coefficient (a marker of riboflavin status where lower EGRac values indicate better riboflavin status); *MTHFR*, methylenetetrahydrofolate reductase (CC; wild type, CT; heterozygous, TT homozygous); SD, standard deviation.

^a^ Participants who consumed foods fortified with B‐vitamins at least once per week.

^b^ Hypertension classified as systolic BP ≥ 140 mmHg and/or diastolic ≥90 mmHg.^39^

^c^ Those taking antihypertensive medications and achieving BP <140/90 mmHg.

* *p*‐Values refer to differences between genotype groups compared using an independent samples *t* test. *χ* ^2^‐test is used for comparison between categorical variables. *p* < 0.05 is considered significant.

### Determinants of hypertension

In this study cohort, the TT genotype was associated with a 71% increased risk of hypertension (odds ratio [OR], 1.71, 95% confidence interval [CI]: 1.14–2.56, *p* = 0.010) after adjustment for significant predictors of BP, namely age (OR: 1.04, 95% CI: 1.02–1.07, *p* < 0.001), male sex (OR: 2.10, 95% CI: 2.36–3.23, *p* = 0.001), BMI (OR: 1.06, 95% CI: 1.02–1.10, *p* = 0.005), antihypertensive drug use (OR: 1.07 95% CI: 0.61–1.87, *p* = 0.818) and a family history of CVD (OR: 1.13, 95% CI: 0.74–1.72, *p* = 0.577; Table 2). Suboptimal riboflavin status, as indicated by EGRac >1.26,^25^ was also associated with an increased risk of hypertension in the same model, OR: 1.97, 95% CI: 1.27–3.05, *p* = 0.002, independent of the *MTHFR* genotype. Regression analysis split by sex revealed that the genotype effect on hypertension appeared to be driven by women (OR: 2.57, 95% CI: 1.24–5.32, *p* = 0.011) compared with a nonsignificant effect in men (OR: 1.51, 95% CI: 0.91–2.50, *p* = 0.110) (Table 2).

**Table 2:** Factors associated with risk of hypertension in study cohort as a whole and stratified by sex

	Cohort as a whole (*n* = 498)				Men (*n* = 300)				Women (*n* = 198)			
	*β*	OR	95% CI	*p* *	*β*	OR	95% CI	*p* *	*β*	OR	95% CI	*p* *
Age (years)	0.041	1.04	1.02–1.07	<0.001	0.030	1.03	1.00–1.06	0.028	0.068	1.07	1.03–1.11	0.001
Male sex	0.740	2.10	2.36–3.23	0.001								
Body mass index (kg/m^2^)	0.055	1.06	1.02–1.10	0.005	0.066	1.07	1.01–1.13	0.019	0.038	1.04	0.98–1.10	0.176
Antihypertensive drug use	0.066	1.07	0.61–1.87	0.818	0.244	1.28	0.64–2.53	0.484	−0.453	0.64	0.22–1.84	0.403
Family history of CVD	0.120	1.13	0.74–1.72	0.577	0.294	1.34	0.80–2.23	0.271	−0.277	0.76	0.37–1.57	0.455
Low riboflavin status^a^	0.677	1.97	1.27–3.05	0.002	0.647	1.91	1.13–3.23	0.016	0.664	1.94	0.88–4.31	0.102
*MTHFR* TT genotype^b^	0.535	1.71	1.14–2.56	0.010	0.411	1.51	0.91–2.50	0.110	0.945	2.57	1.24–5.32	0.011

*Note*: Hypertension is defined as systolic BP ≥140 and/or a diastolic BP ≥90 mmHg.

Abbreviations: BP, blood pressure; CI, confidence interval; CVD, cardiovascular disease; OR, odds ratio.

^a^ Riboflavin biomarker status is determined by the functional assay, erythrocyte glutathione reductase activation coefficient (EGRac). Participants were arbitrarily classed as having ‘lower' or ‘higher' riboflavin status using an EGRac value of 1.26 as a cut‐off point: lower riboflavin status (EGRac <1.26) was compared to higher riboflavin status (EGRac <1.26; reference category).

^b^ Non‐TT (CC, wild type; CT, heterozygous), TT (homozygous), genotypes for the 677 C → T polymorphism in MTHFR; reference category is non‐TT genotype.

* Data analysed by multiple logistic regression with adjustment for other factors in the model. *p* < 0.05 is considered significant.

### BP and central haemodynamic parameters

**Figure 2:**
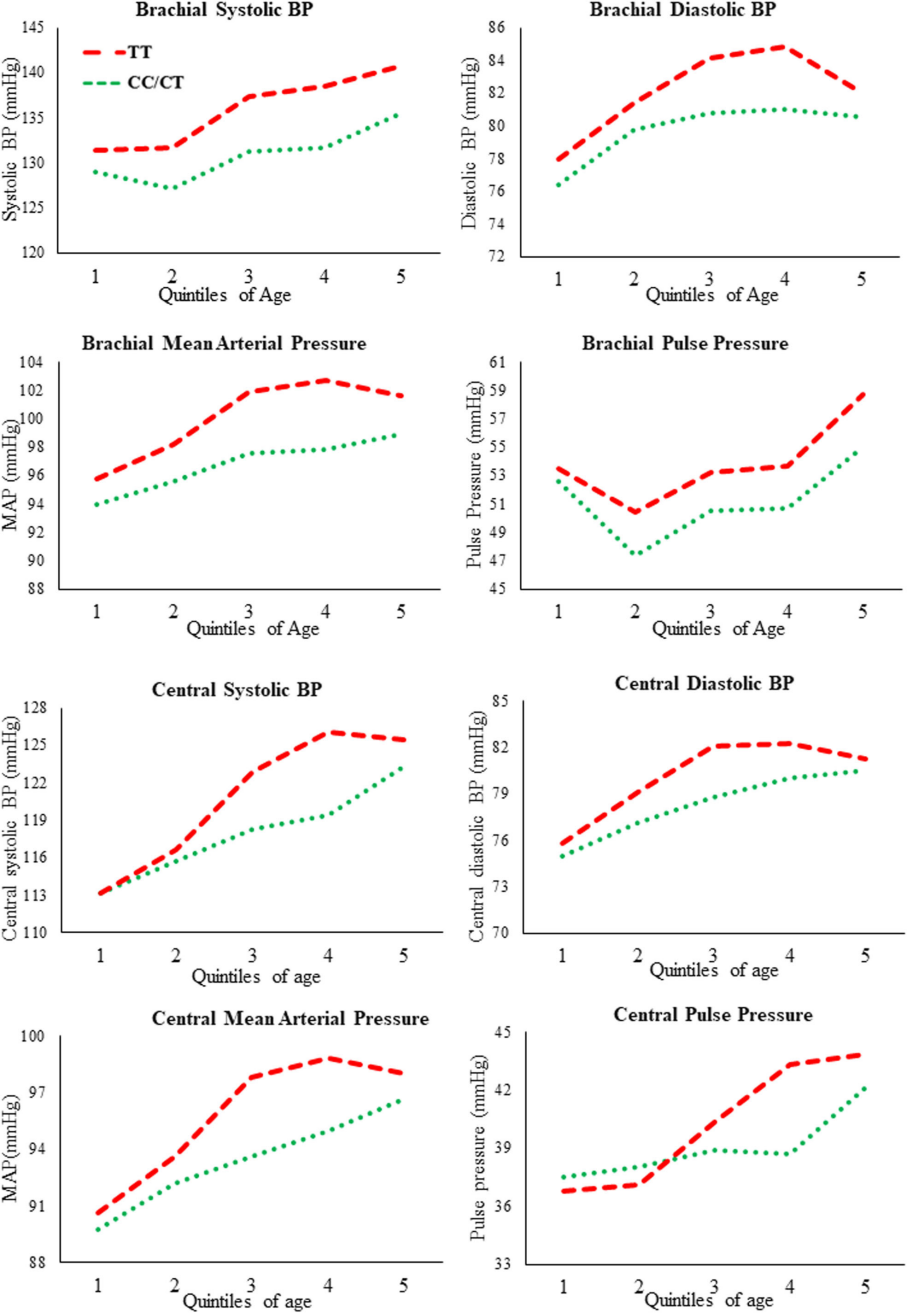
Brachial blood pressure profiles of study participants across quintiles of age,^1^ stratified by *MTHFR* genotype (*n =* 498; CC/CT genotype, *n =* 256; TT genotype, *n =* 242). Data are presented as mean values. (a) Systolic BP; (b) diastolic BP; (c) mean arterial pressure (MAP); and (d) pulse pressure.^1^ Quintiles of age are as follows: <35 years (youngest), 35–42 years, 43–49 years, 50–54 years, >54 years (oldest). MAP is calculated as 1/3 systolic BP plus 2/3 diastolic BP. Pulse pressure is calculated as systolic BP minus diastolic BP.

Brachial systolic and diastolic BP were significantly higher in the TT compared to the non‐TT genotype groups (CT, CC) by 5.5 ± 1.2 and 2.4 ± 0.9 mmHg, respectively (Table 3). In addition, brachial MAP was significantly higher in the TT relative to the non‐TT genotype groups by 3.5 ± 0.9 mmHg. This pattern of elevated brachial pressure in the TT genotype group was evident across the age range of the study (18–65 years), as shown in Figure 2. Similar patterns of elevated pressure in the TT compared to the non‐TT groups were observed, with significant differences between genotype groups in central systolic BP (by 3.1 ± 1.0 mmHg), central diastolic BP (by 1.9 ± 0.8 mmHg), MAP (by 2.5 ± 0.9 mmHg) and PP (by 1.4 ± 0.6 mmHg) observed (Table 3). No genotype effect was evident on measures of PWA or PWV in the cohort as a whole (*n =* 498). When the cohort was split according to normotensive/hypertensive status, the phenotype of elevated BP was still evident. Systolic BP was significantly higher in the normotensive and hypertensive TT individuals when compared to the non‐TT genotype groups (by 1.8 ± 1.3 and 4.1 ± 2.9 mmHg, respectively). In addition, brachial MAP was still significantly higher in the normotensive TT individuals relative to the non‐TT genotype groups. None of the other parameters were statistically significant.

**Table 3:** Blood pressure and central haemodynamic profile of study population by *MTHFR* genotype (*n =* 498)

	*MTHFR genotype*		
	Non‐TT (*n* = 256)	TT (*n =* 242)	*p‐*value*
***Brachial pressure***			
Systolic BP (mmHg)	130.6 (128.9, 132.3)	136.1 (134.4, 137.8)	<0.001
Diastolic BP (mmHg)	79.6 (78.5, 80.7)	82.1 (80.9, 83.2)	0.003
MAP^a^ (mmHg)	96.6 (95.4, 97.8)	100.1 (98.8, 101.3)	<0.001
Pulse pressure^b^ (mmHg)	51.0 (49.7, 52.3)	54.0 (52.7, 55.4)	0.002
*Central pressure*			
Systolic BP (mmHg)	117.8 (116.4, 119.1)	120.9 (119.6, 122.3)	0.001
Diastolic BP (mmHg)	78.2 (77.1, 79.2)	80.1 (79.0, 81.2)	0.011
MAP^b^ (mmHg)	93.3 (92.1, 94.5)	95.8 (94.5, 97.0)	0.004
Pulse pressure^b^ (mmHg)	39.0 (38.2, 39.8)	40.4 (39.5, 41.2)	0.064
***Pulse wave analysis***			
Augmentation pressure (mmHg)	9.0 (8.3, 9.6)	9.6 (9.0, 10.2)	0.112
Augmentation index (%)	22.5 (21.3, 23.7)	23.2 (21.9, 24.4)	0.448
PP amplification	14.6 (14.2, 15.1)	14.7 (14.2, 15.2)	0.791
PP ratio	1.38 (1.37, 1.40)	1.38 (1.36, 1.39)	0.332
*Pulse wave velocity*			
PWV (m/s)^c^	7.47 (7.32, 7.61)	7.59 (7.44, 7.74)	0.201

*Note*: Data are presented as adjusted means (95% CI). All units given as mmHg, unless otherwise stated.

Abbreviations: ANCOVA, analysis of covariance; BP, blood pressure; MAP, mean arterial pressure; PP, pulse pressure; PWV, pulse wave velocity.

^a^ Mean arterial pressure is calculated as 1/3 systolic BP plus 2/3 diastolic BP.

^b^ Pulse pressure is calculated as systolic BP minus diastolic BP.

^c^ For PWV: CC, *n =* 131; CT, *n =* 120; TT, *n =* 236; it was not possible to obtain a measurement of adequate quality in 11 subjects due to attenuation of the pulse signal by subcutaneous fat or difficulty in accessing the position of the artery.

* One‐way ANCOVA adjusting for age, sex, BMI, use of antihypertensive medications and fortified breakfast cereal consumption with Bonferroni post hoc analysis. *p* < 0.05 is considered significant.

### Effect of sex on BP and central haemodynamic parameters

When stratified by sex, the effect of the TT genotype on brachial systolic BP remained evident in both sexes (Supporting Information, Table 2). The phenotype was, however, more pronounced among women, with 9.7 ± 2.0 mmHg higher systolic BP (*p* < 0.001) and 3.4 ± 1.2 mmHg (*p* = 0.005) higher diastolic BP in women with the TT compared to the non‐TT genotypes. Women with the TT genotype were also twice as likely to be classed as hypertensive (i.e., BP ≥ 140/90 mmHg) compared to non‐TT women, 37 versus 18%, respectively (*p* = 0.006, data not shown). For central pressure, the genotype effect was again most pronounced in women, with central systolic BP higher by 5.5 ± 1.7 mmHg (*p* = 0.002) and central diastolic BP higher by 3.7 ± 1.3 mmHg (*p* = 0.004) in the TT compared to the non‐TT genotype women (Supporting Information, Table 2).

When stratified by sex, a genotype effect was observed in some, but not all, PWA measures. Both augmentation pressure and AIx were significantly higher in men with the TT compared to the non‐TT genotypes by 1.2 ± 0.6 mmHg (*p* = 0.034) and 2.3 ± 1.2% (*p* = 0.049), respectively. PWV was significantly faster in women with the TT compared to the non‐TT genotypes by 0.36 ± 0.16 m/s (*p* = 0.043).

## DISCUSSION

We investigated the influence of the *MTHFR* C677T polymorphism on BP and related central haemodynamic parameters in adults aged 18–65 years and observed elevated brachial BP in individuals with the variant *MTHFR* 677TT genotype when compared to those with CC/CT genotypes. Our study provides the first evidence of higher central BP in apparently healthy adults with the TT genotype. Furthermore, our findings indicate that the BP phenotype (both brachial and central BP) associated with this polymorphism was more pronounced in women compared to men. Importantly, suboptimal status of the B‐vitamin riboflavin, the MTHFR cofactor, led to an almost doubling in the risk of hypertension.

In the current study, we report consistently higher brachial systolic and diastolic BP, in addition to MAP and PP, in those homozygous for the common *MTHFR* C677T polymorphism. The higher systolic BP observed in this sample of healthy adults is clinically relevant^27^ and is consistent with our recent observational evidence from over 6000 adults, where we demonstrated that the variant *MTHFR* 677TT genotype is associated with a 42% increased risk of hypertension and predisposes an individual to higher systolic and diastolic BP across adulthood.^11^ The only other study to have investigated the interaction between this polymorphism and BP in a healthy cohort (as opposed to hypertensive or CVD patients) was a study of Japanese men aged 40–59 years; however, the study was limited by a small sample size, with only 14 of the 129 participants carrying the TT genotype.^28^ To the best of our knowledge, this is the first study to report an association between the *MTHFR* C677T polymorphism and PP. PP has recently been associated with all‐cause and CVD mortality in a 6‐year follow‐up of 13,223 Chinese adults aged <65 years,^29^ and although the *MTHFR* genotype was not considered, there is a high frequency of the *MTHFR* 677TT genotype in the Chinese population. Of particular interest in the current study of healthy younger adults, central pressure, as assessed using a range of measures (systolic, diastolic, MAP and PP), was also found to be higher in the variant TT relative to the non‐TT genotype groups, with a significant 3‐mmHg genotype difference observed in central systolic BP. To put this in context, one meta‐analysis of older participants found that a 10‐mmHg increase in central systolic BP was associated with a 9% increased risk of total cardiovascular events.^13^ Furthermore, in one of the studies included in the latter meta‐analysis, the Strong Heart Study involving 2403 participants, a 6‐mmHg higher central PP was found to predict cardiovascular events more strongly than brachial PP, leading the authors to propose central BP as a treatment target for future studies.^30^

PWV, the gold standard measurement of arterial stiffness for predicting future cardiovascular events and mortality^12^, ^13^ and previously associated with BP,^31^ did not differ significantly between the *MTHFR* groups in the overall sample in the current study. Although this finding is contrary to what we had hypothesised, it is consistent with results from three earlier studies in healthy younger^14^, ^32^ and older adults^15^ stratified by the *MTHFR* genotype. When split by sex, however, our results showed for the first time significantly faster PWV among women with the TT compared to the non‐TT genotypes. Because PWV is strongly related to future cardiovascular events and mortality,^12^, ^13^ the findings of the current study provide preliminary evidence that women with the TT genotype may be at particular risk of CVD. Given that the current analysis is based on a relatively small sample of women (*n* = 198), further investigations in larger cohorts of women are required before firm conclusions can be drawn. The largest study conducted to date investigating PWV with respect to the *MTHFR* genotype is a substudy of the China Stroke Primary Prevention Trial (CSPPT),^16^ an RCT investigating the effect of 0.8‐mg folic acid plus enalapril versus enalapril alone in reducing the occurrence of first stroke in hypertensive Chinese patients, stratified by the *MTHFR* genotype.^33^ Similar to the findings of this study, a substudy analysis in CSPPT of 2529 participants reported no difference in PWV between the genotype groups at baseline; however, after the 5‐year intervention period, folic acid was found to reduce PWV, an effect that was greatest in the CC genotype group.^16^ The authors suggested that those with the TT genotype had a higher mechanistic requirement for folic acid; however, the CSPPT did not consider the status of the MTHFR cofactor, riboflavin, which could arguably have had a greater phenotype effect or influenced the response to intervention with folic acid.

Male sex is an important risk factor for both hypertension and CVD; however, it is not widely appreciated that CVD‐related mortality rates are, in fact, higher in women compared to men.^17^, ^19^ A secondary objective of the current study was to examine the effect of sex on BP parameters as this area is largely under‐investigated. In the present study, sex was found to influence both brachial and central pressure, with the greatest phenotype for both measures observed in women. A marked difference of 9.4‐mmHg higher systolic BP was observed in the TT compared to the non‐TT women; a BP difference of this magnitude can be estimated to equate with a 50% higher risk of CVD.^27^ Brachial and central systolic BP are reported to be higher in men compared to women until age 60 years^34^; however, in the current study, both brachial and central BP in the female TT genotype group were similar to BP values measured in the TT and non‐TT genotype men. Although not a primary outcome of the Cardiovascular Risk in Young Finns Study,^32^ the authors reported BP by the *MTHFR* genotype in 1400 young Finnish adults and observed no significant genotype effect; however, only 5.4% of the cohort carried the variant TT genotype, and thus, the study was likely underpowered to detect significant differences. If the phenotype is indeed stronger in women with the variant genotype, it may have important implications for pregnancy. Meta‐analyses have previously linked this polymorphism with an increased risk of hypertension in pregnancy^35^ and pre‐eclampsia,^36^ which affects up to 15% of pregnancies globally and can lead to increased CVD risk in later life for the mother.^6^ Further studies should thus aim to consider the influence of the *MTHFR* genotype on BP and vascular health in pregnancy and in women of reproductive age generally.

Riboflavin was found to be an important modulating factor for BP in this cohort of younger, healthy adults, as suboptimal riboflavin status almost doubled the risk of hypertension after adjustment for other well‐established risk factors. We previously reported an exacerbated genetic risk of hypertension in adults with low or deficient biomarker riboflavin, with a three‐fold risk of hypertension observed for the TT genotype in combination with deficient riboflavin status.^11^ Furthermore, RCTs conducted at this centre have demonstrated that BP is highly responsive to riboflavin supplementation at doses within the dietary range, specifically in adults with the *MTHFR* 677TT genotype. Our trials demonstrated significant reductions of 6–14 mmHg in systolic and 3–8 mmHg in diastolic BP in response to riboflavin intervention, indicating a novel, personalised nutrition approach for the treatment for hypertension in these genetically at‐risk individuals.^7^, ^8^, ^9^ None of these RCTs considered the effect on PWV or alternative measures of vascular health; therefore, further trials to examine the effect of riboflavin intervention on PWV in adults with the TT genotype are needed. The precise mechanism linking the common *MTHFR* C677T polymorphism with hypertension and the modulating effect of riboflavin on BP in these genetically at‐risk individuals remains unexplained but is likely to involve the potent vasodilator, nitric oxide (NO).^6^, ^37^ Both the *MTHFR* genotype and 5‐methyltetrahydrofolate (5‐MTHF), the folate co‐factor generated by the MTHFR‐catalysed reaction, have previously been associated with NO bioavailability in biopsy samples from patients undergoing coronary artery bypass graft (CABG) surgery.^38^ In CABG patients, vascular tissue levels of 5‐MTHF were shown to be lower in adults with the TT genotype (likely as a result of reduced MTHFR activity), which would subsequently affect NO bioavailability and BP. Although not considered in the current study, a noninvasive assessment of endothelial function, such as flow‐mediated dilation, would provide valuable information to help further our understanding of this potential mechanism.

### Strengths and limitations

This study has a number of strengths. It is the largest study of its kind to investigate the effect of the common *MTHFR* C677T polymorphism on both brachial and central pressure. Recruitment was genotype driven and investigated an apparently healthy cohort aged 18–65 years. A comprehensive noninvasive investigation of vascular health, including PWV, considered to be the gold standard noninvasive measurement for arterial stiffness, was conducted; to the best of our knowledge this is the first time these measures have been reported in a sample stratified by *MTHFR* genotype and sex. Furthermore, all vascular measures were conducted by the same researcher to minimise inter‐operator variability. The study was, however, not without limitations. Although the study was powered to investigate the effect of genotype on BP parameters, it was not powered for the secondary analysis for sex effects, and therefore, future studies are required to confirm the current findings with regard to sex differences. In addition, further biomarker data, in particular measures of folate and other B‐vitamins and intermediates of the one‐carbon network of pathways, were not available but could provide further insights into the role of one‐carbon metabolism and this gene–nutrient interaction in BP. Future studies with additional measures of endothelial function, such as flow‐mediated dilation, could provide additional insights into the relationship between the TT genotype and vascular function and related effects on NO bioavailability.

## CONCLUSION

In conclusion, this is the first study to show that both brachial and central BP are significantly higher in healthy adults aged up to 65 years with the variant *MTHFR* 677TT genotype and that the BP phenotype and effect on PWV are more pronounced in women. Moreover, low biomarker status of riboflavin was found to be independently associated with hypertension. Given the high prevalence of the *MTHFR* C677T polymorphism globally, these findings have important implications for the prevention and treatment of hypertension in adults worldwide with this genetic risk factor.

## AUTHOR CONTRIBUTIONS

All authors contributed equally.

## CONFLICTS OF INTERESTS

The authors declare that they have no conflict of interest.

## PEER REVIEW

The peer review history for this article is available at https://publons.com/publon/10.1111/jhn.13061

## Supporting information

Supporting information.
