# Supplementary material for: Impact of the MTHFR C677T polymorphism on blood pressure and related central haemodynamic parameters in healthy adults
Source: J Hum Nutr Diet. 2022 Jul 27;35(4):689–700. doi: 10.1111/jhn.13061 (PMC9541256; doi:10.1111/jhn.13061)
Supplement: Supplementary file 1 — Supporting information. [file JHN-35-689-s001.docx]

**Supplementary table 1.** Antihypertensive drugs taken by participants at time of sampling (*n* 76)

|  |  | ***MTHFR* genotype** | |
| --- | --- | --- | --- |
|  |  | **Non-TT**  **(*n* 41)** | **TT**  **(*n* 35)** |
| *Drug combination* | |  |  |
| 1 BP medication | | 33 (80) | 21 (60) |
| 2 BP medications | | 5 (12) | 12 (34) |
| 3 BP medications | | 3 (7) | 2 (6) |
| *Drug Class* | |  |  |
| ACE inhibitor | | 19 (58) | 22 (63) |
| CCB | | 12 (36) | 15 (43) |
| ß blocker | | 8 (24) | 3 (9) |
| ARB | | 3 (9) | 5 (14) |
| Diuretic | | 2 (6) | 3 (9) |

Values presented as *n* (%).

ACE inhibitor, angiotensin-converting enzyme inhibitor; ARB, angiotensin II receptor blockers; CCB, calcium channel blocker.

**Supplementary table 2.** Blood pressure and central hemodynamic profile of study participants, stratified by *MTHFR* genotype and sex (*n* 498)

|  | ***Male*** | | |  | ***Female*** | |  |  |
| --- | --- | --- | --- | --- | --- | --- | --- | --- |
|  | **CC/CT**  **(*n* 162)** | | **TT**  **(*n* 138)** | ***P* value^*^** | **CC/CT**  **(*n* 94)** | **TT**  **(*n* 104)** | ***P* value^*^** | |
| *Brachial pressure* |  | |  |  |  |  |  | |
| Systolic BP (mmHg) | 133.7 (131.6,135.7) | | 137.2 (134.9,139.4) | 0.026 | 125.2 (122.3,128.0) | 134.9 (132.1,137.6) | <0.001 | |
| Diastolic BP (mmHg) | 80.3 (78.8, 81.7) | | 81.9 (80.3, 83.5) | 0.146 | 78.6 (77.0, 80.3) | 82.1 (80.4, 83.7) | 0.005 | |
| MAP^†^ (mmHg) | 98.1 (96.5, 99.6) | | 100.3 (98.7,102.0) | 0.052 | 94.2 (92.2, 96.1) | 99.7 (97.8, 101.5) | <0.001 | |
| Pulse pressure^‡^ (mmHg) | 53.4 (51.9, 54.9) | | 55.2 (53.6, 56.8) | 0.120 | 46.5 (44.4, 48.7) | 52.8 (50.7, 54.9) | <0.001 | |
| *Central pressure* |  | |  |  |  |  |  | |
| Systolic BP (mmHg) | 118.5(116.8,120.1) | | 120.8(119.0,122.6) | 0.077 | 116.1(113.8,118.5) | 121.6(119.3,123.8) | 0.002 | |
| Diastolic BP (mmHg) | 79.3 (78.0, 80.7) | | 80.2 (78.7, 81.6) | 0.437 | 76.3 (74.5, 78.0) | 80.0 (78.3, 81.7) | 0.004 | |
| MAP^†^ (mmHg) | 94.1 (92.6, 95.6) | | 95.7 (94.0, 97.3) | 0.154 | 91.9 (90.0, 93.9) | 96.0 (94.1, 97.9) | 0.004 | |
| Pulse pressure^‡^ (mmHg) | 38.6 (37.6, 39.6) | | 40.1 (39.1, 41.2) | 0.046 | 39.2 (37.7, 40.7) | 41.1 (39.7, 42.5) | 0.218 | |
| *Pulse wave analysis* |  | |  |  |  |  |  | |
| Augmentation pressure (mmHg) | | 7.1 (6.4, 7.9) | 8.3 (7.5, 9.1) | 0.034 | 11.5 (10.4, 12.6) | 11.8 (10.8, 12.9) | 0.736 | |
| AIx (%) | 18.1 (16.5, 19.7) | | 20.4 (18.7, 22.1) | 0.049 | 29.0 (27.0, 31.0) | 27.7 (25.8, 29.6) | 0.353 | |
| PP amplification | 16.0 (15.4, 16.5) | | 15.7 (15.1, 16.3) | 0.482 | 12.5 (11.7, 13.3) | 13.3 (12.6, 14.1) | 0.181 | |
| PP ratio | 1.42 (1.41, 1.44) | | 1.40 (1.38, 1.42) | 0.056 | 1.33 (1.31, 1.34) | 1.34 (1.32, 1.36) | 0.384 | |
| *Pulse wave velocity* |  | |  |  |  |  |  | |
| PWV (m/s)^§^ | 7.67 (7.48, 7.85) | | 7.68 (7.48, 7.89) | 0.861 | 7.11 (6.89, 7.34) | 7.47 (7.25, 7.69) | 0.043 | |

Data presented as adjusted means (95% CI). All units given as mmHg, unless otherwise stated.

^*^ One-way ANCOVA adjusting for age, BMI, use of anti-hypertensive medication and consumption of fortified breakfast cereals with

Bonferroni post-hoc analysis. PWV also adjusted for consumption of fortified breakfast cereals. Chi-square test used to detect differences in categorical variables. *P*<0.05 considered significant. Values within a row with different superscript letters (a, b) indicate significant differences between groups.

^†^ Mean arterial pressure (MAP) calculated as 1/3 systolic BP plus 2/3 diastolic BP.

^‡^ Pulse pressure calculated as systolic BP minus diastolic BP.

^§^ For PWV in the male cohort, CC/CT *n* 159; TT *n* 134. For PWV in the female cohort CC/CT *n* 92; TT *n* 102.

AIx, augmentation index; BP, blood pressure; MAP, mean arterial pressure; PP, pulse pressure; PWV, pulse wave velocity.
